# Case Series: Evidence of Borderzone Ischemia in Critically-Ill COVID-19 Patients Who “Do Not Wake Up”

**DOI:** 10.3389/fneur.2020.00964

**Published:** 2020-09-23

**Authors:** Letitia Pirau, Lauren Ottenhoff, Craig A. Williamson, Shahid N. Ahmad, Rafael Wabl, Andrew Nguyen, Laura Faiver, Venkatakrishna Rajajee

**Affiliations:** ^1^Department of Neurology, University of Michigan, Ann Arbor, MI, United States; ^2^Department of Neurosurgery, University of Michigan, Ann Arbor, MI, United States

**Keywords:** COVID-19 (2019-nCoV), encephalopathy, coma, magnetic resonance imaging, cerebral ischemia, borderzone infarction

## Abstract

This article describes the clinical course, radiological findings, and outcome of two patients with the novel 2019 coronavirus disease (COVID-19) who remained comatose for a prolonged duration following discontinuation of all sedation. These two male patients, one aged 59-years and another aged 53-years, both with a history of hypertension and neurologically intact on admission, developed worsening COVID-19 associated acute respiratory distress syndrome (ARDS). Both required benzodiazepine, opioid, neuromuscular blockade, therapeutic anticoagulation, and vasopressor infusions in addition to renal replacement therapy. Echocardiography demonstrated normal chamber size and systolic function in both cases. Each patient demonstrated only trace flexion to pain 7–10 days following discontinuation of all sedation. Magnetic Resonance Imaging on both patients demonstrated multifocal lesions on diffusion weighted imaging with apparent diffusion coefficient correlate in bilateral middle/anterior cerebral artery borderzones, and no large-vessel occlusion or severe stenosis. In both patients, continuous electroencephalography demonstrated no seizures. Neither patient had any documented period of sustained hypotension (mean arterial pressure <60 mmHg) or hypoxia (SpO_2_ <90%). Ninety days following initial presentation, the 59-years-old man was oriented, with fluent speech and able to ambulate with assistance, while the other 53-years-old man was at home and independent, undertaking the basic activities required by daily living. We conclude that critically-ill COVID-19 patients with prolonged coma following sedation discontinuation may demonstrate imaging features of ischemic injury in borderzone regions despite the absence of documented sustained hypotension or hypoxia. However, substantial neurological recovery is possible despite these findings.

## Introduction

Critically-ill patients with the novel 2019 coronavirus infection (COVID-19) have demonstrated a variety of neurological complications, such as anosmia, encephalopathy, large vessel ischemic stroke, and encephalitis (1–4). Some patients present with acute encephalopathy and develop acute respiratory distress syndrome (ARDS) (4, 5). Intubated COVID-19 patients frequently require sedation for the management of hyperactive delirium, and also require prolonged use of sedative, analgesic, and neuromuscular blocking (NMB) medication infusions for the management of acute hypoxic respiratory failure, which has led to shortages in the United States (6).

An important problem encountered in the care of COVID-19 patients who require intubation for ARDS and acute hypoxic respiratory failure is prolonged unresponsiveness following discontinuation of sedation (7). While prolonged sedation is sometimes expected in this context, especially with the use of agents, such as Midazolam and some opioid agents in patients with renal dysfunction (8), and some patients have remained poorly responsive for weeks following discontinuation of all sedating agents, often in the context of improvement in their pulmonary status. The persistence of coma beyond a week following discontinuation of all forms of sedation, suggests the presence of either prolonged encephalopathy or acute brain injury, including acute ischemic injury, and leads to questions about etiology as well as long-term neurological prognosis. While sustained periods of severe hypoxia, hypotension, or low cardiac output may result in hypoxic-ischemic injury, in our experience, some COVID-19 patients have remained comatose in the absence of such documented events. While the distinction between reversible encephalopathy and structural brain injury has critical prognostic implications, there is no published data on whether prolonged unresponsiveness in intubated COVID-19 patients implies the presence of irreversible injury, and whether neurological recovery can occur.

In this report, we describe the clinical course, radiological findings, and outcome of two such COVID-19 patients, initially intubated for ARDS while neurologically intact, who demonstrated prolonged unresponsiveness following discontinuation of sedation, with evidence of borderzone ischemia on Magnetic Resonance Imaging (MRI) of the brain.

## Description of Cases

### Case 1

**Figure 1:**
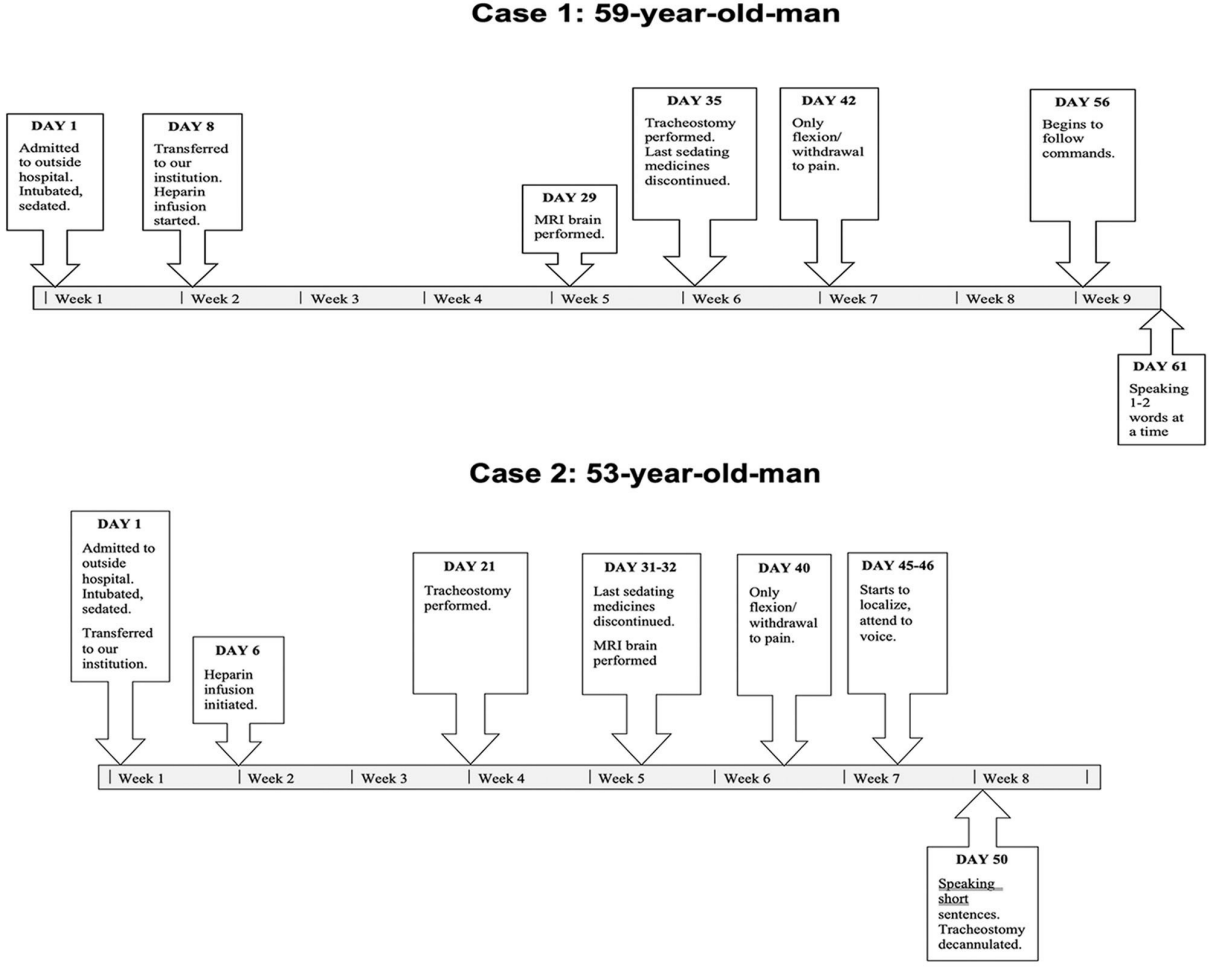
Timeline of events for both cases.

A 59-years-old man with hypertension and rheumatic heart disease, no significant family or psychosocial history, presented with acute hypoxic respiratory failure from COVID-19 infection and rapidly developed ARDS. A timeline of events is shown in Figure 1.

He was neurologically intact on admission to an outside facility. He subsequently required intubation, deep sedation, high positive end expiratory pressure (PEEP), NMB, prone positioning, vasopressor infusion, and renal replacement therapy (RRT). He was transferred to our institution 8 days following admission to the outside facility. He was empirically anticoagulated with an unfractionated Heparin infusion shortly following admission for an elevation in D-Dimer (8.47 mg/L). He was sedated with Midazolam, Lorazepam, Dexmedetomidine, Fentanyl, and Oxycodone. Echocardiography revealed only left ventricular hypertrophy, with normal left and right ventricular chamber size and systolic function. All sedating medications were progressively weaned down and discontinued 35 days following admission.

**Figure 2:**
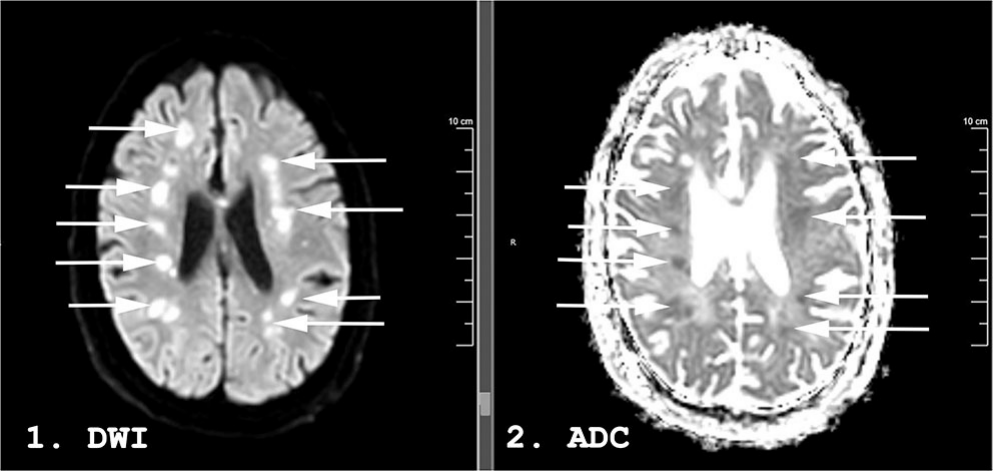
Magnetic resonance imaging (MRI) performed 31 days following admission on a 59-years-old man with COVID-19. Diffusion Weighted Imaging (DWI) in panel 1 and apparent diffusion coefficient (ADC) in panel 2. Hyperintense lesions are seen in the bilateral middle/anterior cerebral artery (MCA-ACA) borderzones on DWI (arrows). Some demonstrate a hypointense ADC correlate (arrows) while others are isointense on ADC, suggesting an acute to subacute timeframe.

A week after the discontinuation of all sedating medication, his best neurological examination was a weak flexion/withdrawal response to pain. Laboratory, CT, and continuous electroencephalography data at this time are shown in Table 1. An MRI of the brain scan performed 29 days following initial presentation demonstrated multifocal regions of hyperintensity on Diffusion Weighted Imaging (DWI) with a corresponding hypointensity on apparent diffusion coefficient (ADC) in the bilateral middle cerebral artery (MCA)—anterior cerebral artery (ACA) borderzones (Figure 2).

**Table 1:** Laboratory, CT, and continuous electroencephalography evaluation performed ~10 days following discontinuation of all sedation, during the time period when both patients remained comatose.

**Test performed**	**Patient 1 (59-M)**		**Patient 2 (53-M)**	
	**Value**	**Days from initial presentation**	**Value**	**Days from initial presentation**
Admission pO_2_ (mmHg)	90	8	175	1
Admission P/F ratio	90	8	350	1
Lowest recorded pO_2_ (mmHg)	53	19	51	3
Highest PEEP (cmH_2_O)	18	9	18	8
Sodium (mmol/L)	139	46	140	32
Potassium (mmol/L)	4	46	3.0	32
Chloride (mmol/L)	100	46	98	32
Bicarbonate (mmol/L)	27	46	32	32
Blood urea nitrogen (mg/dL)	53	46	53	32
Creatinine (mg/dL)	3.6	46	3.93	32
White blood cell count (K/uL)	13.3	46	13.3	32
Hemoglobin (g/dL)	8.2	46	7.9	32
Platelet count (K/uL)	315	46	473	32
Magnesium (mg/dL)	2.0	46	1.6	32
Phosphorous (mg/dL)	3.5	46	2.5	32
Ionized calcium (mmol/L)	1.34	51	1.14	34
Ammonia (umol/L)	23	23	Not collected	
Procalcitonin (ng/mL)	0.67	35	2.81	35
Blood cultures	Negative	45	Negative	34
Urine culture	*Candida albicans*	46	Negative	27
Ferritin (ng/mL)	1554.8	9	1380	1
Lactate dehydrogenase (LDH) (IU/L)	453	9	843	1
C-Reactive protein (mg/dL)	25.0	9	42.1	1
D-Dimer (mg/L)	8.47	9	>35	5
CT non-contrast of the brain	No acute intracranial process	34	No acute intracranial process	21
Continuous electroencephalography	Disorganized and slow background, occasional frontal dominant diffuse rhythmic delta (FIRDA), occasional independent left more than right temporal polymorphic delta slowing, occasional left temporal sharp waves	32	Disorganized and slow background with minimal reactivity and runs of FIRDA, suggesting moderate encephalopathy. Relatively greater attenuation over the posterior quadrant and right hemisphere, rare focal right temporal slowing	32

*Inflammatory markers (C-reactive protein, Ferritin, and Lactate Dehydrogenase) reflect admission values at our institution while the D-Dimer test in the table represents the value that triggered the initiation of anticoagulation*.

No significant intra-cranial large-vessel disease was present on Gadolinium enhanced MR imaging of the intracranial arteries. Carotid ultrasound revealed only minimal bilateral plaque at the bifurcation without significant stenosis. He underwent tracheostomy 35 days following admission. No period of sustained (>15 min) hypotension [mean arterial pressure (MAP) <60 mmHg] or hypoxia (SpO_2_ <90%) was documented. Blood pressure was monitored using both non-invasive blood pressure (NIBP) measurements as well as invasive arterial monitoring in the radial artery for the duration of the patient's stay in ICU. The lowest measured MAP was 56 mmHg, resolved within 15 min following titration of vasopressor.

Sixty-one days following initial presentation, at the time of last follow-up, he had significantly improved. He was following simple appendicular commands, able to state his name, the month, and the year, able to resist with his right arm and leg, and move his left side with gravity eliminated. Ninety days following his initial presentation, he was in a subacute rehabilitation center, oriented and able to converse fluently, able to ambulate short distances with maximal assistance. Further functional improvement was anticipated.

### Case 2

A 53-years-old man with hypertension and chronic pain and opioid use, no significant family or psychosocial history, was neurologically intact on admission and required intubation the day of admission for COVID-19 associated ARDS and increasing oxygen requirements. A timeline of events is shown in Figure 1.

He was transferred to our institution on the same day he was admitted. He subsequently required deep sedation, high PEEP, NMB, prone positioning, vasopressor infusion, and RRT. He was empirically anticoagulated with an unfractionated Heparin infusion 6 days after admission for an elevation in D-Dimer (>35 mg/L). He was sedated with Midazolam, Lorazepam, Dexmedetomidine, Fentanyl, and Oxycodone. Echocardiography revealed only left ventricular hypertrophy, with normal left and right ventricular chamber size and systolic function. Tracheostomy was performed 21 days following admission. All sedating medications were progressively weaned down, and all infusions discontinued 26 days following admission, while enteral Lorazepam and Oxycodone were discontinued 31 days from admission, respectively.

**Figure 3:**
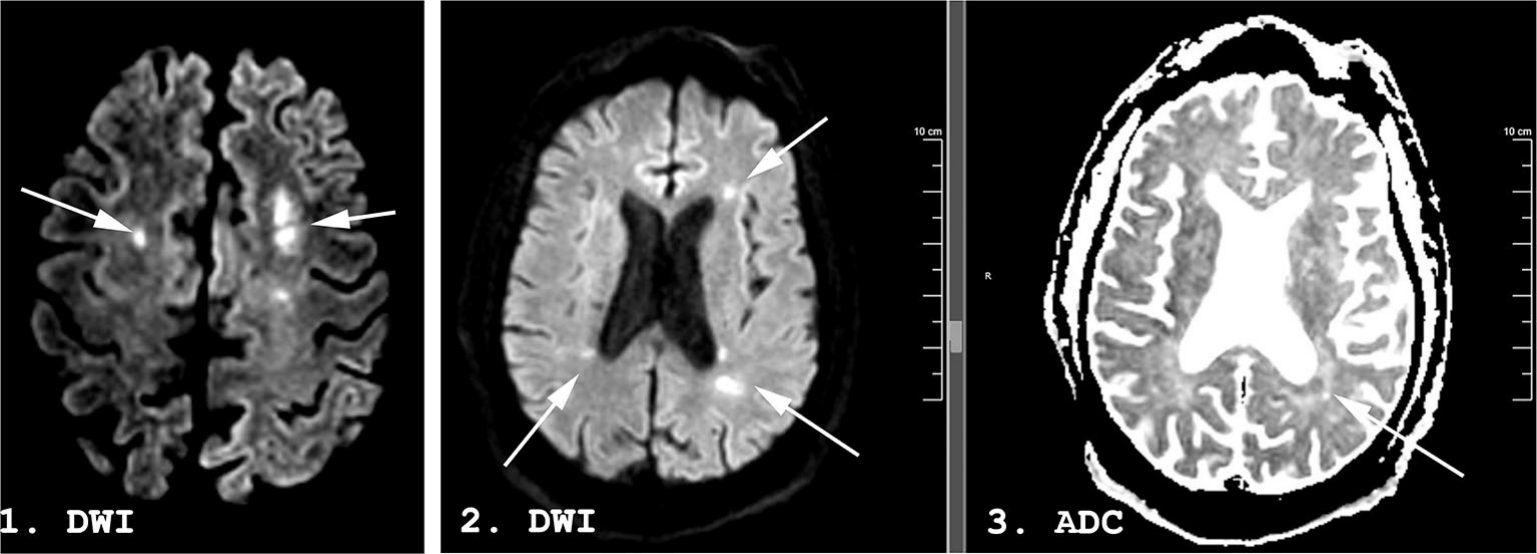
A 53-years-old man with COVID-19. Magnetic resonance imaging (MRI) performed 29 days following admission. Diffusion Weighted Imaging (DWI) in panels 1 and 2, apparent diffusion coefficient (ADC) in panel 3. Hyperintense lesions (arrows) are seen in the bilateral middle/anterior cerebral artery (MCA-ACA) borderzones on DWI. Some demonstrate a hypointense ADC correlate (arrow) while others are isointense on ADC, suggesting an acute to subacute timeframe.

A week following discontinuation of all sedating medication his best neurological examination was eye opening to noxious stimuli and a weak flexion/withdrawal response to pain. Laboratory, CT, and continuous electroencephalography data at this time are shown in Table 1. MRI brain performed 31 days following initial presentation demonstrated multifocal DWI lesions in the left > right MCA-ACA borderzones (Figure 3). Some of these lesions demonstrated hypointensity on ADC while others were iso-intense, suggesting an acute to subacute timeframe.

No significant intra-cranial large-vessel disease was present on Gadolinium enhanced MR imaging of the intracranial arteries. Carotid ultrasound was unremarkable bilaterally, without significant plaque or narrowing. No period of sustained (>15 min) hypotension (MAP <60 mmHg) or hypoxia (SpO_2_ <90%) was documented. Blood pressure was monitored using both non-invasive blood pressure (NIBP) measurements as well as invasive arterial monitoring in the radial artery for the duration of the patient's ICU stay. The lowest measured MAP was 51 mmHg, resolved within 15 min following titration of vasopressor.

Forty-five days following initial presentation, he demonstrated more purposeful movement bilaterally and tracked to voice. Fifty days after admission, he was awake, attentive, and speaking short sentences fluently, and oriented only to the location (“hospital”), with a right pronator drift. The tracheostomy was decannulated on day 50. Ninety days following initial presentation he was at home, oriented, able to converse fluently, and perform all basic activities of daily living within the home.

## Discussion

In this case-series, we have described an important clinical problem, the critically-ill COVID-19 patient intubated for respiratory failure, with prolonged unresponsiveness off sedation. While prolonged encephalopathy or unresponsiveness may occur in critically ill patients with a variety of diagnoses, this problem has recently been of specific interest in the context of the COVID-19 pandemic (7).

The differential diagnosis in this situation typically includes a prolonged toxic-metabolic encephalopathy (often without a specific etiology identified), vs. acute structural brain injury. While these patients certainly demonstrate severe toxic-metabolic encephalopathy at different time-points of their ICU stay, the most important information from this report may be that structural brain injury can occur. The presence of very similar borderzone infarctions in both the cases described suggests ischemic injury.

The most likely etiologies include a period of hemodynamic compromise resulting in hypoperfusion, thromboembolism, and a cardiac source. Echocardiography and blood cultures were unremarkable, and imaging of the intra- and extra- cranial vasculature did not reveal stenosis or occlusion. The bilateral nature of these ischemic borderzone lesions, along with the presence of ischemic lesions in internal borderzones (9), without lesions scattered within multiple vascular territories, may favor global hypoperfusion as an etiology. However, there was no documented period of severe sustained hypotension (MAP <60 mmHg) in either case and echocardiography demonstrated normal left and right ventricular systolic function.

A pro-thrombotic state is well-documented in COVID-19 patients (10, 11). Specifically, there have been reports of large-vessel acute ischemic strokes in younger COVID-19 patients (2). Consistent with practice at many centers, therapeutic anticoagulation was started in both cases relatively quickly following admission, in response to an elevation in D-Dimer. However, a thrombo-embolic etiology certainly remains possible despite the use of anticoagulation.

Most of the published data on neurological complications of COVID-19 have focused on patients who present with neurological symptoms, rather than the more common presentation with respiratory failure, complicated by a profound and prolonged disorder of consciousness. Large-vessel ischemic strokes have been described in COVID-19 patients without other risk factors (2). Encephalopathy on presentation is common (12–14). There are reports of imaging consistent with encephalitis and, possibly, parenchymal invasion (3, 15).

MRI scans are challenging to perform in critically-ill COVID-19 patients, because of patient instability as well as concerns about transmission to healthcare staff. The only case-series of critically-ill COVID-19 patients in whom MRI was performed described 27 patients in whom 12 demonstrated positive findings (16). These findings were largely non-specific and included cortical restricted diffusion, leptomeningeal enhancement, and punctate cortical blooming artifact, in addition to individual patients with ischemic stroke or venous sinus thrombosis.

Of note, is the fact that this study was not focused exclusively on patients with prolonged coma. Another study of 37 COVID-19 patients (32 of whom critically-ill) revealed several patterns of abnormalities on MRI, including multi-focal regions of acute ischemia and mesial temporal lobe hyperintensity (17). Critically-ill patients were more likely to demonstrate punctate hemorrhagic lesions. This study was also not focused on patients with prolonged unresponsiveness off sedation.

The important take-away points from this report for intensivists could be that scrupulous attention to maintaining adequate blood pressure and oxygenation at all times is necessary, since these patients may be vulnerable to brain injury at a threshold and duration of hypotension/hypoxia not typically thought to result in permanent structural injury. These two patients had a history of hypertension, albeit treated, and may have therefore been particularly vulnerable to hemodynamic compromise. These ischemic findings may also bolster the argument for therapeutic anticoagulation in critically-ill COVID-19 patients, however, this is controversial (10, 11, 18). Most importantly, the substantial delayed neurological recovery in both patients, despite the prolonged duration of coma and evidence of ischemic injury on MRI, suggests that patience and continued supportive care, rather than a premature determination of poor prognosis, may be appropriate in some patients.

This is a small case-series, and larger observational studies are necessary to determine the spectrum of neurological injury as well as predictors of long-term outcomes in this population. Both patients were transferred to us from other facilities. While we did have access to medical records from the outside hospital, including a summary of vital signs, it is possible that more serious periods of hemodynamic compromise occurred in both cases that were not documented in the records available to us. We did not perform cerebrospinal fluid (CSF) testing in either patient to determine the presence of the virus within the brain. This was primarily because MR imaging suggested ischemic injury and was not consistent with viral encephalitis, as has been reported (3, 15), but also because of the uncertainties around reverse transcription polymerase chain reaction (RT-PCR) testing of CSF for viral ribonucleic acid (RNA). While a positive test in CSF may simply represent contamination from blood, a negative test might not exclude parenchymal invasion. We also did not investigate a possible central nervous system inflammatory or autoimmune response to COVID-19, since imaging was not consistent with auto-immune encephalitis.

## Patient Perspective

At the time of the 90-days follow-up, both patients and their families expressed satisfaction with their decision to persist with medical treatment and prolonged life support. Both acknowledged the limitations in their functional ability and quality of life but expressed their hope that the progressive improvement in functional ability would continue.

## Conclusion

Critically-ill COVID-19 patients who remain comatose for prolonged periods following discontinuation of sedation may demonstrate features of hypoperfusion injury in borderzone regions despite the absence of sustained severe hypotension or hypoxia. However, substantial neurological recovery is possible despite these findings.

## Data Availability Statement

The original contributions presented in the study are included in the article/supplementary material, further inquiries can be directed to the corresponding author/s.

## Ethics Statement

The studies involving human participants were reviewed and approved by University of Michigan Institutional Review Board (HUM00180961). Written informed consent for participation was not required for this study in accordance with the national legislation and the institutional requirements. Written informed consent was obtained from the individuals or legally authorized representatives for the publication of any potentially identifiable images or data included in this article.

## Author Contributions

VR, LP, LO, and CW conceived of the study. LP, LO, and VR collected the data for the manuscript and wrote the draft of the manuscript. CW, SA, RW, AN, and LF edited the manuscript and collected the additional data. All authors have reviewed and approved the final version of the manuscript.

## Conflict of Interest

The authors declare that the research was conducted in the absence of any commercial or financial relationships that could be construed as a potential conflict of interest.
